# Supplementary material for: The rights and wrongs of rescaling in population genetics simulations
Source: PLoS Genet. 2026 Aug 5;22(8):e1012261. doi: 10.1371/journal.pgen.1012261 (PMC13489532; doi:10.1371/journal.pgen.1012261)
Supplement: Supplementary file 1 [file pgen.1012261.s001.docx]

**S1 Table** : Estimated *B* values in simulations with various scaling factors (*C*) and simulated population sizes (*N*_sim_). In all cases, expected *B* = 0.74. 100 replicates were simulated for simulations with *N*=10^6^ and 10 replicates were simulated for simulations with *N*=5×10^6^ diploid individuals. The assumed mutation rate (*u*) and recombination rate (*r*) are listed per site/generation. The length (*L*) of the simulated region was either 1 Mb or 5 Mb and is specified below. Selected sites experienced purifying selection with a fixed selective disadvantage (2*N_sim_s*=100). *ML* denotes the map length in the simulated population in Morgans, assuming no crossover interference. SE represents the standard deviation of the means across the simulated replicates. Note that simulations with *N*_sim_=25,000 and 50,000 did not reach completion in a reasonable time and thus have been omitted.

| **Parameters** | **Sample size** | **stat** | ***C*=100** | ***C*=200** | ***C*=500** | ***C*=1000** |
| --- | --- | --- | --- | --- | --- | --- |
| ***From Figure 1:*** | | | | | |  |
|  |  |  | ***ML*=1** | ***ML*=2** | ***ML*=5** | ***ML*=10** |
|  |  |  | ***N*sim=10k** | ***N*sim=5k** | ***N*sim=2k** | ***N*sim=1k** |
| *N_e_*=10^6^ *u*=3×10^-9^  *r*=1×10^-8^ *L*=1 Mb | Entire population | mean | 0.737 | 0.730 | 0.682 | 0.550 |
|  |  | SE | 0.009 | 0.008 | 0.009 | 0.007 |
|  | 100 genomes | mean | 0.730 | 0.723 | 0.675 | 0.544 |
|  |  | SE | 0.009 | 0.008 | 0.009 | 0.007 |
|  | 10 genomes | mean | 0.737 | 0.730 | 0.682 | 0.549 |
|  |  | SE | 0.010 | 0.009 | 0.010 | 0.008 |
| ***Larger natural population size; theta and rho are increased in proportion*** | | | | | |  |
|  |  |  | ***ML*=1** | ***ML*=2** | ***ML*=5** | ***ML*=10** |
|  |  |  | ***N*sim=50k** | ***N*sim=25k** | ***N*sim=10k** | ***N*sim=5k** |

| *N_e_*=5×10^6^ *u*=3×10^-9^ *r*=1×10^-8^  *L*=1 Mb | Entire population | mean | - | - | 0.725 | 0.692 |
| --- | --- | --- | --- | --- | --- | --- |
|  |  | SE | - | - | 0.003 | 0.004 |
|  | 100 genomes | mean | - | - | 0.718 | 0.685 |
|  |  | SE | - | - | 0.003 | 0.004 |
|  | 10 genomes | mean | - | - | 0.726 | 0.692 |
|  |  | SE | - | - | 0.003 | 0.005 |
| ***Larger natural population size; theta and rho are preserved*** | | | | | | |
|  |  |  | ***ML*=0.2** | ***ML*=0.4** | ***ML*=1** | ***ML*=2** |
|  |  |  | ***N*sim=50k** | ***N*sim=25k** | ***N*sim=10k** | ***N*sim=5k** |
| *N_e_*=5×10^6^ *u*=0.2×3×10^-9^  *r*=0.2×1×10^-8^  *L*=1 Mb | Entire population | Mean | - | - | 0.739 | 0.728 |
|  |  | SE | - | - | 0.003 | 0.002 |
|  | 100 genomes | mean | - | - | 0.731 | 0.720 |
|  |  | SE | - | - | 0.003 | 0.002 |
|  | 10 genomes | mean | - | - | 0.736 | 0.726 |
|  |  | SE | - | - | 0.003 | 0.002 |
| ***Larger natural population size; theta and rho are preserved; length of the region is increased; the ratio U/M is preserved (M is the map length in Morgans)*** | | | | | | |
|  |  |  | ***ML*=1** | ***ML*=2** | ***ML*=5** | ***ML*=10** |
|  |  |  | ***N*sim=50k** | ***N*sim=25k** | ***N*sim=10k** | ***N*sim=5k** |
| *N_e_*=5×10^6^ *u*=0.2×3×10^-9^  *r*=0.2×1×10^-8^  *L*=5 Mb | Entire population | mean | - | - | 0.726 | 0.691 |
|  |  | SE | - | - | 0.001 | 0.001 |
|  | 100 genomes | mean | - | - | 0.719 | 0.684 |
|  |  | SE | - | - | 0.001 | 0.001 |
|  | 10 genomes | mean | - | - | 0.726 | 0.690 |
|  |  | SE | - | - | 0.001 | 0.001 |
