# Supplementary material for: The rights and wrongs of rescaling in population genetics simulations
Source: PLoS Genet. 2026 Aug 5;22(8):e1012261. doi: 10.1371/journal.pgen.1012261 (PMC13489532; doi:10.1371/journal.pgen.1012261)
Supplement: Supplementary file 2 [file pgen.1012261.s002.docx]

**S1 Fig**: Expected time to conditional loss of neutral mutations (relative to 2*N*_e_ generations) as a function of the scaling factor plotted using equation A7.
